# Study on the effect of 3,5,6,7,8,3′,4′-heptamethoxyflavone in *Fructus Aurantii* by regulating intestinal aquaporin in body fluids

**DOI:** 10.3389/fphar.2025.1544570

**Published:** 2025-05-19

**Authors:** Ting-Ting Bai, Ya-Ting Xie, Quan Wan, Jin-Lian Zhang

**Affiliations:** ^1^ Department of Pharmacy, Jiangxi University of Chinese Medicine, Nanchang, China; ^2^ Mongolian College of Medicine, Inner Mongolia Medical University, Hohhot, China; ^3^ Affiliated Hospital of Inner Mongolia Minzu University, Tongliao, China

**Keywords:** 3,5,6,7,8,3′,4′-heptamethoxyflavone, *Fructus aurantii*, aquaporins, body fluid, intestinal tract

## Abstract

**Background:**

*Fructus aurantii* (FA) can cause the drying of body fluids, although the specific mechanism of this process remains unclear. FA is used to promote stagnation and eliminate flatulence in traditional Chinese medicine. 3,5,6,7,8,3′,4′-heptamethoxyflavone is the active metabolite in FA, and it contributes to the drying process. This paper presents an investigation into the underlying mechanisms of the effect of 3,5,6,7,8,3′,4′-heptamethoxyflavone on body fluids through regulating the Aquaporin pathway.

**Methods:**

Human small intestinal epithelial cells (FHs74lnt), human colon histiocytes (CCD018Co), a normal mouse, and an AQP3 knockout mouse, were used in the study. Indicators included the water consumption, diet, and fecal water content of the mice, as well as pathological changes in the small intestinal and colon tissues and the relative expression of AQP3, AQP5, AQP7, and AQP11 mRNA in those tissues, and protein expression. ALD and ADH hormone levels, and the AQP3 upstream receptor genes AVPR1 and AVPR2 were also used as indicators for examination.

**Results:**

3,5,6,7,8,3′,4′-heptamethoxyflavone has a strong desiccating impact on body fluids, causing an increase in water intake and a rise in the water content of feces. Additionally, animal experiments have suggested a connection to histological damage in the colon and small intestine, including lymphocyte infiltration, mucosal laminae breakage, and local ulceration. Additionally, the expression of the proteins Aquaporin 3, 5, 7, and 11 in the intestinal tissues may be regulated by 3,5,6,7,8,3′,4′-heptamethoxyflavone. This metabolite also increases the expression of upstream receptor genes, allowing them to bind more easily to antidiuretic hormone and aldosterone, such as arginine vasopressin receptors 1 and 2. A number of changes in water intake and fecal water content were observed in response to 3,5,6,7,8,3′,4′-heptamethoxyflavone in experiments conducted on Aquaporin3−/− mice. The control of water secretion and absorption in the body is thus impacted by alternating the Aquaporin 3 water channels.

**Conclusion:**

3,5,6,7,8,3′,4′-heptamethoxyflavone may affect upstream receptor genes, including AVPR1 and AVPR2, and promote their binding to ALD and ADH, which in turn affects the opening and closing of the AQP3 water channel to regulate water secretion and absorption in the body.

## 1 Introduction

Based on the principles of traditional Chinese medicine (TCM), dryness is considered a countermeasure to dampness, which can cause various symptoms such as abdominal distension, reduced appetite, altered taste, nausea, vomiting, lethargy, diarrhea, and a thick and greasy coating on the tongue. Dryness is associated with conditions such as dry mouth, nose, and throat, thirst, dry or cracked skin, and decreased urine output, and dry stools often accompany excessive dryness or insufficient treatment. Clinical indicators used to assess the “dryness” characteristics of a medication include water intake, diet, salivary flow rate, urine volume, and stool consistency (Jinlian, 2018).

*Fructus Aurantii* (FA), derived from the cultivated unripe fruit of *Citrus aurantium* L., belongs to the Rutaceae family and is widely used as a herbal remedy in TCM (Jinlian, 2018; Jing et al., 2018; Junbin et al., 2024; Di et al., 2025). Traditionally, is it regarded as a regulator of “Qi”, for alleviating abdominal distension, and promoting smooth digestion (Songhong et al., 2023; Shupei et al., 2023; Yangbing et al., 2025). 3,5,6,7,8,3′,4′-heptamethoxyflavone (HMF), the main pharmaceutical metabolite in FA, is a flavonoid with strong biological activity (Wenhui et al., 2024), characterized by low polarity, a planar structure, and the presence of multiple methoxy groups. Previous studies have shown that it has anti-inflammatory, antibacterial, antioxidant, and anti-artherosclerotic properties, and can assist in weight loss and lipid-lowering, along with neuroprotective effects (Pengcheng et al., 2015; Yu et al., 2022; Meizi et al., 2025). Research has also revealed that FA has desiccative properties, and can damage body fluids, resulting in intestinal dryness and constipation (Jing et al., 2018; Jiangling et al., 2022; Tingting et al., 2023; Haiou et al., 2025). HMF is one of the primary metabolites causing dryness in FA. Dryness, which is a typical indicator of an organism’s deficiency in water, is a central idea in Chinese medical thought. Body dryness is associated with symptoms such as a dry mouth, nose, and throat, dry or cracked skin, dry feces, and reduced urine production. It can also increase the risk of benign intestinal adenomas, colorectal cancer, and intestinal polyps in cases of severe intestinal injury (Yating et al., 2023). A loss of body fluids can lead to high blood pressure, heart-related accidents, and renal and cardiovascular problems, among other conditions (Jinlian, 2018; Jing et al., 2023). In recent years, despite increasing research into natural plant drugs by the medical profession, much remains to be understood about the metabolite causing dryness. Study of the changes in body fluids and water regulation and balance caused by HMF has emerged as a new potential research direction in this field.

Aquaporins (AQPs) are integral membrane proteins that selectively mediate the efficient transport of water molecules (Charlotte et al., 2022). They exhibit similar structures and functions across various types and are responsible for regulating water movement both within and outside cells, making them essential for sustaining human life (Li et al., 2005; Zuoyi et al., 2022). Recent clinical studies have clarified the distribution of AQPs in the gastrointestinal tract and their main functions (Laforenza et al., 2012; Shengyun et al., 2023; Dandan et al., 2023). Detecting AQPs can serve as an indicator for the development and regression of diseases (Ricanek et al., 2015). Furthermore, research has demonstrated the presence of AQPs in diverse animal species, highlighting their broad physiological functions such as promoting gastrointestinal motility and protecting the integrity of the intestinal mucosal barrier. These findings have shown promising potential for the application of AQPs in various areas.

The regulation of fluids is indeed important as dryness can lead to fluid imbalances and blood loss. The gut, being the primary organ responsible for water absorption and excretion, contains a significant number of AQPs, which play a vital role in maintaining water balance within the intestine and are implicated in the onset and progression of various disorders. However, it remains unclear how the dryness caused by HMF affects the normal metabolism of intestinal tissues and fluids. Therefore, further investigation is required to understand the mechanisms by which HMF influences water channel proteins. Moreover, gaining a deeper understanding of the functions of AQPs can open up new possibilities for targeting water channel proteins through inhibitors. These inhibitors may potentially serve as novel drug targets for the treatment of numerous human diseases related to water balance and fluid regulation.

## 2 Materials and methods

### 2.1 Ethics statement

This study adhered to the guidelines provided by the Institutional Animal Ethical Committee of Jiangxi University of Chinese Medicine. Prior to carrying out the research, the study protocol received approval from the Animal Ethics Committee (Ethics number: JZLLSC20210018).

### 2.2 Materials

Aquaporin 3 (AQP3), Aquaporin 5 (AQP5), Aquaporin 7 (AQP7), and Aquaporin 11 (AQP11) were purchased from Affinity Biosciences (Jiangsu, China). Glyceraldehyde-3-phosphate dehydrogenase (GAPDH) was purchased from Cell Signaling Technology. Secondary antibodies were acquired from Cell Signaling Technology. The following chemicals and solvents were procured from specific suppliers: acetonitrile, chromatographic grade methanol (ACS, 63Y1706TE, 73C1812PR), anhydrous ethanol (Batch No. 1911261), ethyl acetate (Batch No. B2101141), hexane (Batch no. 1608271), analytical purity methanol (Batch No. 2010051), and petroleum ether (Batch No. 2102011) were purchased from Xilong Ltd., while deuterated chloroform was purchased from Ningbo Zuiying Chemical Technology Co. Additionally, we purchased HMF (Batch No.CHB-Q-097, ≥98%) from Chengdu Kromax Biotechnology Co.

### 2.3 Drug preparation

*Citrus aurantium* L., No. ZQ20200719, was purchased from the cultivation base located in Shangrao City, Jiangxi Province. The plant material was verified by Professor Ge from the Teaching and Research Department of Chinese Medicine Resources, Jiangxi University of Chinese Medicine (National Pharmacopoeia Committee, 2015). For *in vivo* experiments, HMF was dissolved in pure water and administrated to mouse. As for *in vitro* experiments, HMF was mixed with serum-free methyl sulfoxide (DMSO) and sonicated in an ultrasonic bath until fully dissolved (Jinna et al., 2021). The drug solution was then filtered using a 0.22 μm micropore filter (Chen et al., 2021). The resulting liquid had a final concentration of 500 μg/mL and was stored at −20°C.

A quantity of 500 g of FA was weighed, powdered, and sieved. It was then placed in a round-bottomed flask, and an appropriate amount of zeolite was added. For the initial step, 600 mL of anhydrous ethanol was added to the flask. The mixture was left to infuse for 30 min and subsequently refluxed for 1 h. The same amount of anhydrous ethanol was added for the second reflux step, and reflux was continued for 1 h. The resulting filtrates from both reflux steps were collected, combined, and concentrated to obtain the infusion. For the HMF control solution, a weight of 0.43°mg of HMF was measured and placed in a volumetric flask with a capacity of 2 mL. The solution was fixed with methanol and passed through a 0.22 μm microporous filter membrane to obtain the control solution.

To dissolve the crude extract of FA, 300 mL of water was added. The extraction was repeated twice using ethyl acetate (300 mL) each time. The resulting extracts were collected, and the ethyl acetate layers were combined. The solution was spin-dried to recover the ethyl acetate. The obtained extract was precisely weighed at 0.017 g and placed in a 2 mL volumetric flask. It was fixed with methanol and passed through a 0.22 μm microporous membrane. The solution was then set aside. For further processing, 200 mL of methanol was added to dissolve the sample. Subsequently, 250 mL of n-hexane was added for sample extraction. The n-hexane phase was collected, and the solution was spin-dried and set aside. The sample was loaded onto an open silica gel column (200–300 mesh, 20 nm × 200 cm). Elution was performed using 1,000 mL of ethyl acetate-petroleum ether (1:6), followed by 1,000 mL of ethyl acetate-petroleum ether (1:3).

The HMF control was taken and precisely weighed. It was dissolved in a specific quantity of methanol to prepare a solution with a concentration of 1.25 μg/mL. The solution was then passed through a 0.45 μm microporous membrane for filtration. Approximately 0.2 g of the crude powder from each *C. aurantium* tablet was weighed in a 50 mL conical flask with a stopper. To the conical flask, 50 mL of methanol was added. The flask was weighed, and the extract was refluxed for 1.5 h. After cooling, the flask was weighed again, and the reduced weight was compensated with methanol. The mixture was thoroughly shaken and filtered. The resulting filtrate was then subjected to another round of filtration. A volume of 10 mL of the filtrate was transferred to a 25 mL volumetric flask. The flask was fixed with methanol, shaken well, and an appropriate amount of the solution was passed through a 0.45°μm microporous filter membrane.

### 2.4 HPLC conditions

The separation was carried out on a SuperLu C18 column (250 × 4.6 mm) with the mobile phase eluted with a gradient of acetonitrile (A): 0.1% formic acid (B): 0–5 min: 5%–50% A; 5–15 min: 50%-70%A. The flow rate was 1 mL/min with an injection volume of 10 μL, and the detection wavelength was 328 nm at 30°C.

### 2.5 Animals and groups

C57BL/6 (wild-type, WT) (18 ± 2 g) mouse and AQP3 knockout (AQP3 KO) mouse were purchased from Yakang Biotechnology Co. (SCXK (苏) 2018–0027). The animal facility maintained a cleanliness level of 10,000, utilizing a positive pressure system with a differential pressure above 10 Pa. The ventilation rate was set at 15 times per hour, with an air flow rate of 0.1–0.2 m/s. The ambient temperature was maintained at 24°C ± 2°C, and the humidity level was kept at 55% ± 5%. Bedding, feeding, and drinking water were changed daily to maintain sterility and provide appropriate care for the animals.

After 1 week of acclimatization feeding, C57BL/6 were divided randomly into 5 groups of 10 mice each. AQP3 knockout and wild-type mice were divided into 3 groups of 3 mice each. The HMF low, medium, and high dosage groups were gavaged with 0.3, 0.6, and 0.9 mg/kg of HMF test solution, respectively. The 2020 edition of Chinese Pharmacopoeia (Part 1) stipulates that the dosage of *C. aurantium* should be 3–10 g, and the HMF concentration was calculated as 0.1% (Ren et al., 2018). FA (1.3 mg/kg) was calculated according to a normal adult weight of 60 kg, combined with the mouse-human surface area ratio (Ren et al., 2018).

### 2.6 General indicator monitoring

The water and food intake of the mouse was assessed both prior to and following drug administration. Measurements were recorded over a period of 24 h at four specific time points: 1 day before, 3 days, 6 days, 9 days, and 12 days after drug administration. Each cage was provided with a predetermined quantity of water and feed, which were weighed and given to the mouse at 8:30a.m. every morning. The remaining water and feed were then measured at 8:30a.m. the following morning.

### 2.7 Fecal water content testing

The water content of the feces was measured before and after drug administration. Fecal samples were collected at specific time points: 1 day before, 3 days, 6 days, 9 days, and 12 days after drug administration. The wet weight of each sample was determined using an analytical balance. Subsequently, the samples were placed in an electric thermostatic oven set at 105°C for 48 h until a constant weight was achieved. The dried samples were then weighed again using an analytical balance to determine the dry weight.

### 2.8 HE staining

The small intestine and colon tissues were fixed using a 4% paraformaldehyde solution to immobilize them. Once appropriately sized tissues were obtained, the surface was washed to remove any residual paraformaldehyde, followed by dehydration and embedding in paraffin wax. The embedded tissues were then sectioned into slices with a thickness of 4–5 μm. The resulting slides were collected and subjected to baking at 60°C for 1–2 h to enhance tissue adherence. Subsequently, the slides underwent dewaxing using xylene, followed by rehydration using different concentrations of ethanol. To visualize the nuclei and cytoplasm, the slides were stained according to the instructions provided in the hematoxylin-eosin staining kit.

### 2.9 Immunohistochemistry staining

Immunofluorescence staining was performed to assess the expression and activity of AQP3, AQP5, AQP7, and AQP11 in tissue cells. The tissue sections were initially covered with a 3% bovine serum albumin (BSA) solution, which was then sealed and left at room temperature for 30 min. After removing the blocking solution, the sections were incubated overnight at 4°C with a drop of phosphate-buffered saline (PBS) and the corresponding primary antibody (1:1,000). Then, the sections were washed three times with PBS for 5 min each to remove excess primary antibody. For tissue coverage, the primary antibody corresponding to the species was dropwise added, followed by incubation for 50 min at room temperature. This primary antibody was labeled with horseradish peroxidase (HRP). To visualize the staining, the sections were treated with the 3,3′-diaminobenzidine (DAB) color solution, which produced a brown color reaction (Li et al., 2020). After further washing, the sections were observed under a Nikon fluorescence microscope to visualize the immunofluorescent signals.

### 2.10 Cell culture

FHs74lnt and CCD018Co cell lines were cultured in a humidified incubator at 37°C with 5% CO_2._ The culture medium consisted of 10% fetal bovine serum (FBS) and a mixture of 90% Dulbecco’s Modified Eagle Medium (DMEM) without sodium pyruvate, 10% FBS, and 1% triple antibodies (penicillin, gentamicin, kanamycin). To evaluate the effects of HMF on cell viability, FHs74lnt and CCD018Co were seeded into 96-well plates. Different concentrations of HMF (40, 20, 10, 5, 2.5, and 1.25 μg/mL) were added to the wells, along with 10% FBS and 1% penicillin/streptomycin (P/S) in DMEM medium. The cells were then incubated for 48 h. Next, 20 μL of 3-(4,5-dimethylthiazol-2-yl)-2,5-diphenyltetrazolium bromide (MTT) solution at a concentration of 5 mg/mL was added to each well and incubated for 4 h at 37°C. The supernatant was discarded, and 150 μL of dimethyl sulfoxide (DMSO) was added to each well. The plate was then shaken for 10 min to dissolve the formazan crystals. Finally, the optical density (OD) value at 570 nm was measured using an enzyme marker to assess cell viability and metabolic activity (Nga et al., 2020).

### 2.11 Assay for activity levels of aldosterone and antidiuretic hormone

The activity levels of aldosterone (ALD) and antidiuretic hormone (ADH) in mouse small intestine and colonic tissue were measured using commercially available enzyme-linked immunosorbent assay (ELISA) kits (Jiangsu Zeyu Biotechnology Co., Jiangsu, China).

### 2.12 Real-time polymerase chain reaction

Total RNA was extracted from the samples using the Total RNA Purification kit (Sangon Biotech). The RNA was then reverse transcribed into complementary DNA (cDNA) using a transcriptase kit (Thermo Fisher Scientific) (Li et al., 2021a). To analyze the gene expression of pyroptosis, specific primers were designed and used for amplification. The sequences of these primers can be found in the Supplementary Tables S1, S2. Quantification of gene expression was performed using the 2^−ΔΔCT^ method, which allows for relative quantification. The expression levels of the target genes were normalized to the expression of glyceraldehyde-3-phosphate dehydrogenase (GAPDH), which served as the internal reference gene.

### 2.13 Western blot

Protein extraction was performed from the aortas of mouse in each experimental group. The protein concentrations were quantified using a bicinchoninic acid (BCA) kit. To analyze the extracted proteins, 20 μg of each sample was loaded onto a 10% sodium dodecyl sulfate-polyacrylamide gel electrophoresis (SDS-PAGE) gel. The proteins were separated based on their molecular weight during electrophoresis. Subsequently, the separated proteins were transferred from the gel to polyvinylidene fluoride (PVDF) membranes (Millipore, IPVH00010). To prevent non-specific binding, the PVDF membranes were blocked for 2 h. Next, the membranes were incubated overnight at 4°C with primary antibodies (1:1,000), suitably diluted to ensure optimal specificity and sensitivity. Following the primary antibody incubation, the membranes were washed three times with PBS to remove any unbound antibodies. Subsequently, the membranes were incubated with secondary antibodies (1:5,000) for 1 h at room temperature, allowing for the detection of protein bands of interest. Finally, the protein expression levels of the different experimental groups were compared (Li et al., 2021b).

### 2.14 Statistical analysis

The statistical analysis was performed using GraphPad Prism 8.0 (GraphPad Software). All data are presented as Mean ± SD. Student’s t-tests were used to assess the differences between two groups. For multiple comparisons, the one-way analysis of variance (ANOVA) test was employed. Spearman’s correlation analysis and the chi-square (x^2^) test were conducted to evaluate the correlation between different gene pairs. The log-rank test was used to assess survival differences. All statistical analyses were two-sided, and *P* < 0.05 was considered to indicate statistical significance.

## 3 Results

### 3.1 Identification and analysis of HMF

**FIGURE 1:**
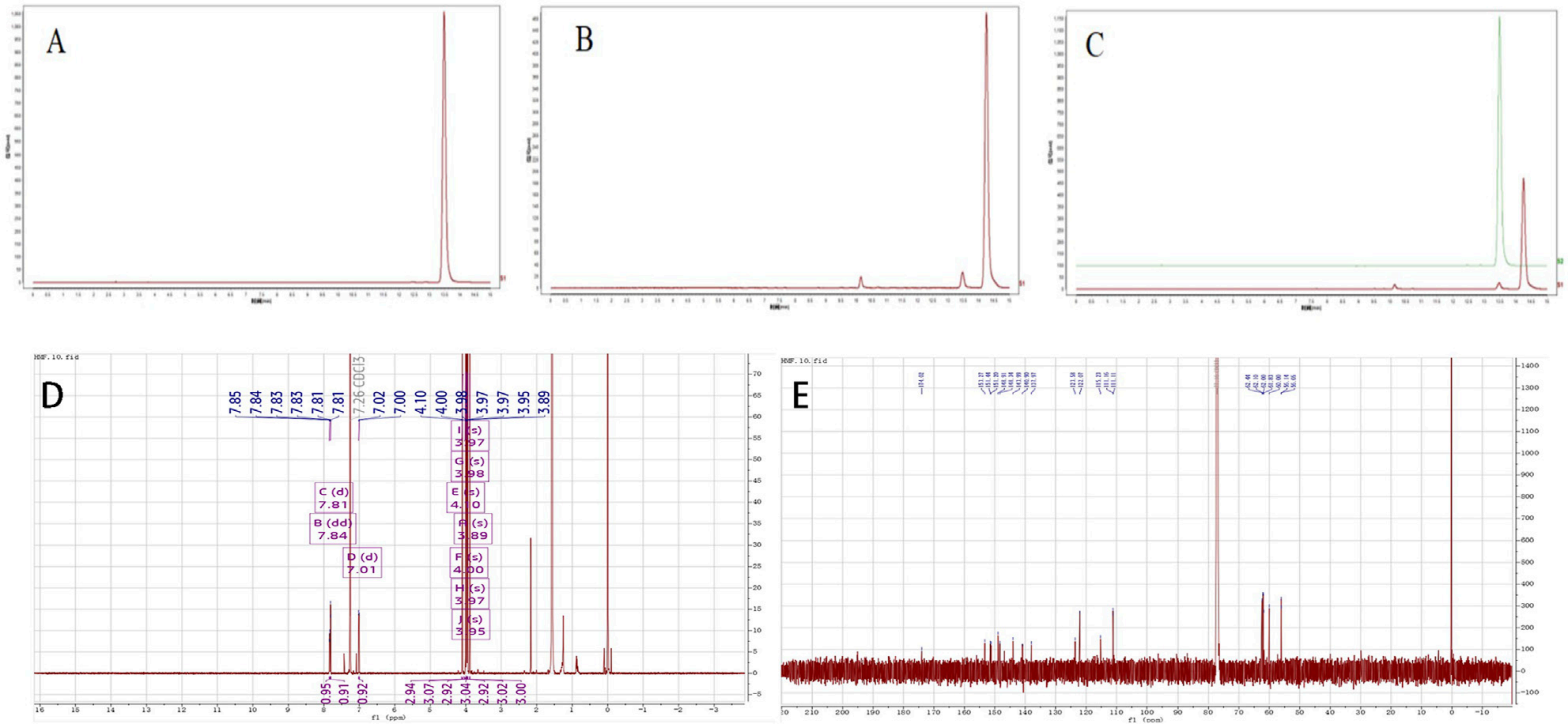
**(A–C)** The analysis of HPLC results **(A)** Standardised product; **(B)** Sample of FA; **(C)** Standardised product and sample); **(D, E)** Uclear magnetic resonance (NMR) identification analysis, **(D)** 1H-NMR analy- sis results, **(E)** 13C-NMR analysis results.

The HPLC analysis revealed that the HMF control exhibited a peak time of 13.489 min and a peak area of 772,086 AU (Figure 1A). A liquid phase analysis was performed on the purified FA sample, leading to a peak time of 13.477 min and a peak area of 209,953 AU (Figure 1B). These peak times were used to quantify the HMF content in the FA, which was determined to be HMF (Figure 1C).

The structural identification of the isolated powder was conducted using nuclear magnetic resonance (NMR) spectroscopy, the analysis revealed a molecular formula of C_22_H_24_O_9_, a melting point of 129°C–131°C, and ESI-MS m/z values of [M + H]^+^ 432.58 and [M + Na]^+^454.20. The UV-8000S showed absorption peaks at 243–277°nm and 308–362°nm. The 13C-NMR (150 MHz. CDCl3) signals indicated alkene carbon signals at δ 174.02, 153.27, 151.44, 151.2, 148.91, 148.34, 143.99, 137.97, 140.9, 123.58, 122.07, and 115.23. The 1H-NMR (600 MHz, CDCl3) spectrum exhibited peaks at δ 7.85–7.83 (dd, J = 2.04, 2.1, 1H), 7.81 (d, J = 1.98, 1H), 7.02–7.00 (d, J = 8.58,1H), 4.1 (s, 3H), 4.00 (s, 3H), 3.98 (s, 3H), 3.97 (s, 3H), 3.97 (s, 3H), 3.95 (s, 3H), 3.89 (s 3H); by 4.1 (s, 3H), 4.00 (s, 3H), 3.98 (s, 3H), 3.97 (s, 3H), 3.97 (s, 3H), 3.95 (s, 3H), and 3.89 (s, 3H), from which it was further determined that the metabolite structure contained seven methoxyl groups. The proton signals in the hydrogen spectra [δ 7.85–7.83 (dd, J = 2.04, 2.1, 1H), 7.81 (d, J = 1.98, 1H), 7.02–7.00 (d, J = 8.58, 1H)] indicated the metabolite to be an ABX-coupled system, likely a proton signal on the B ring of the flavone, shown in the Figures 1D–E; Table 1 for details. The obtained data agreed with the literature (Han et al., 2010). Based on these findings, the isolated and purified metabolite was identified as HMF.

**TABLE 1:** ^13^CNMR,^1^H-NMR analysis.

^13^C-NMR			^1^H-NMR		
Carbon spectrum Data	Spectral library data	Placement	Hydrogen spectrum data	Placement	Peaks
174.02	174	C-4	7.81	H-2′	d
153.27	153.2	C-4′	7.84	H-6′	dd
151.44	151.4	C-7	7.01	H-5′	d
151.2	151.1	C-2	3.97	6-OMe	
148.91	148.9	C-3′	3.98	3′-OMe	
148.34	148.3	C-9	4.1	7-OMe	
143.99	144	C-5	3.89	8-OMe	
137.97	138	2C, C-6,8	4	5-OMe	
140.9	140.9	C-3	3.97	4′-OMe	
123.58	123.6	C-1′	3.95	3-OMe	
122.07	122.1	C-6′			
115.23	115.2	C-10			
111.16	111.2	C-5′			
111.11	111.1	C-2′			
62.44	62.4	C			
62.1	62	C			
62	61.9	C			
61.83	61.7	C			
60	59.9	C			
56.14	56.1	C			
56.05	56	OCH3			

### 3.2 Determination of sample content

**FIGURE 2:**
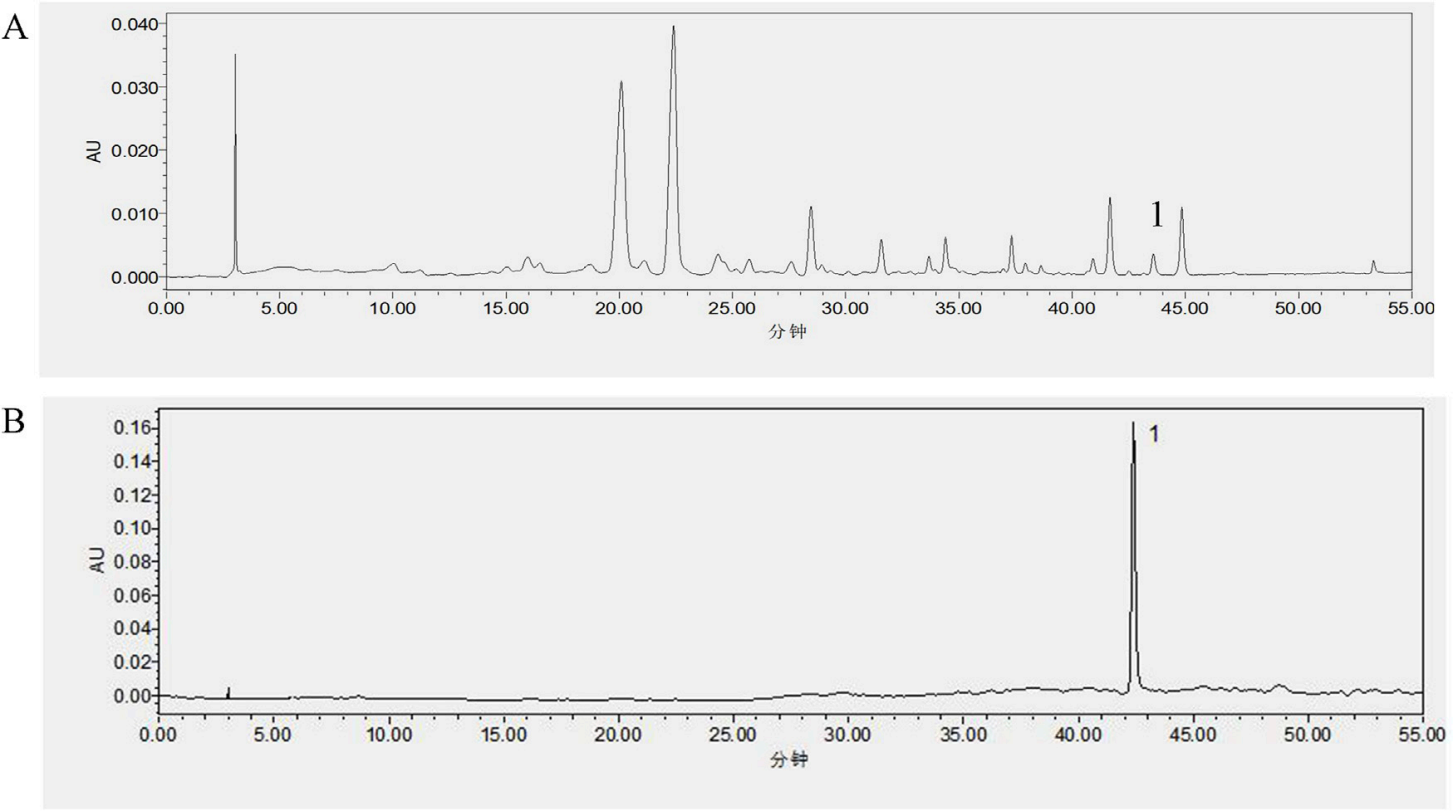
Results of content determination, **(A)** FA; **(B)** 3′,4′,3,5,6,7,8-heptamethoxyflavone.

The peak areas were determined based on the established chromatographic conditions described in Section 2.4 Sample volumes of 2 μL, 4 μL, 8 μL, 10 μL, 16 μL, and 20 μL from the control solution (see Section 2.3) were injected into the system. A standard curve was generated with the amount of control solution on the horizontal axis and the corresponding peak area on the vertical axis. The regression equation for HMF was calculated to be Y = 2089.4X-1155.6, with a linear range from 2.5 to 25 μg and a correlation coefficient R^2^ = 0.9985. The content of HMF in the raw product of FA was calculated to be 0.0104% from the regression analysis (Figures 2A, B).

### 3.3 Effects of 3′,4′,3,5,6,7,8-heptamethoxyflavone on body fluids, small intestine and colon tissues in mouse

**FIGURE 3:**
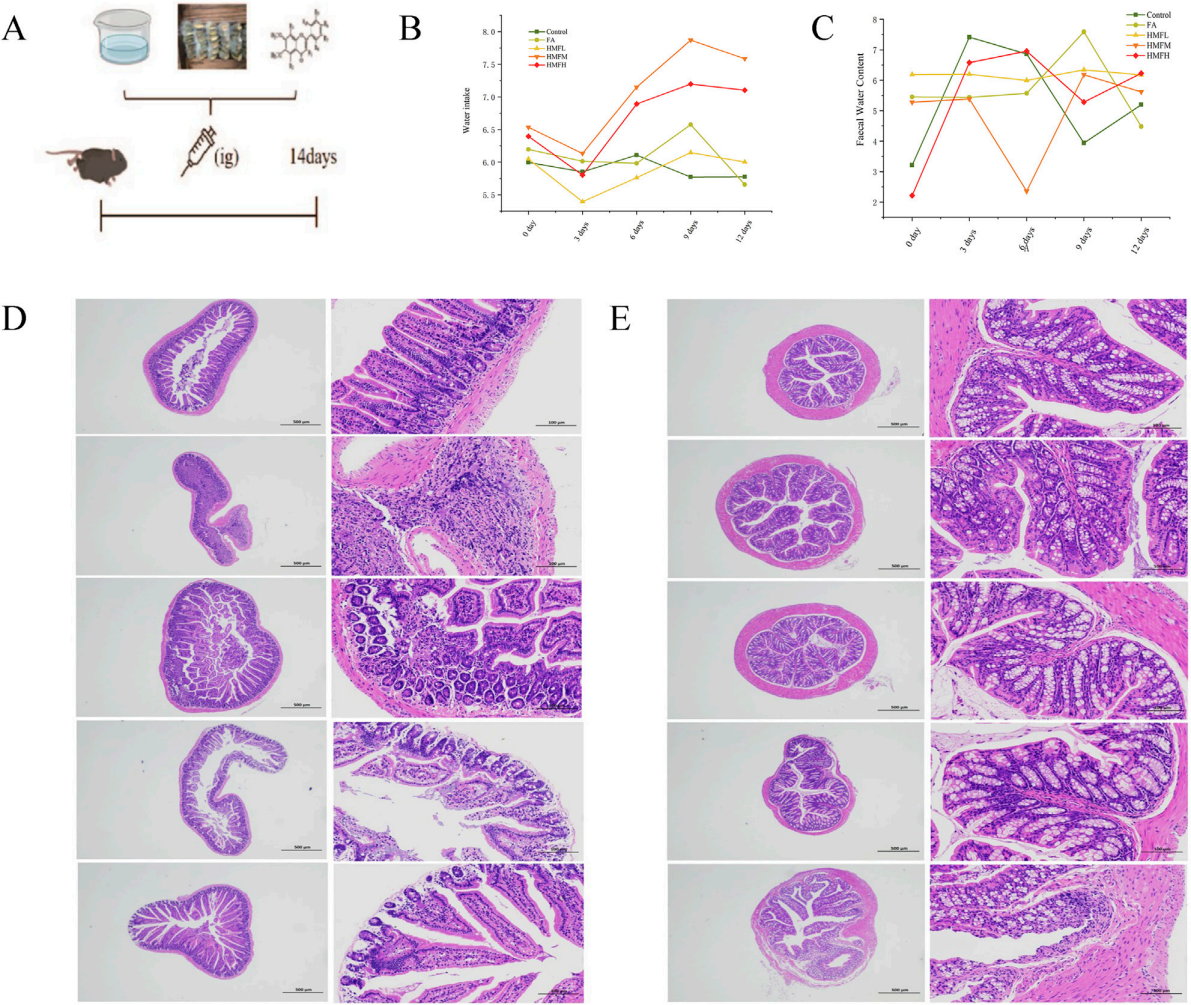
**(A)** Animal expeiment flow chart; **(B)** Water intake of mouse; **(C)** Faecal water content for mouse, **(D, E)** Pathological staining results was analyzed in all groups in the small intestine and colon (n = 3). Note: Results of histopathological staining of all the small intestine on the left and the colon intestine histopathological staining on the right.

The study showed a decrease in fecal water content in the HMFL, HMFM, and HMFH groups compared to the control group (Figures 3A–C). The treatment groups induced increased water intake in normal mouse compared to the control group, indicating a specific impact on fluid balance. Histological analysis of small intestinal tissues in the control group showed no abnormalities, whereas the FA, HMF-High (HMFH), HMF-Medium (HMFM), and HMF-Low (HMFL) groups exhibited multiple ulcers, mucosal muscle layer damage, rupture, and intestinal gland necrosis. These changes were accompanied by hyperplastic connective tissue and significant lymphocyte infiltration (Figure 3D). In the colonic tissue analysis, no apparent abnormalities were observed in the control, FA, HMFL, or HMFM groups, but the HMFH group displayed ulceration, mucosal muscle layer rupture, and lymphocyte infiltration (Figure 3E). Previous research reported that rats with constipation had significantly higher levels of AQP3 protein expression in their intestinal tissues, which affected water reabsorption in the colon and reduced fecal water content.

### 3.4 Effect of 3′,4′,3,5,6,7,8-heptamethoxyflavone on mouse water channel protein

**FIGURE 4:**
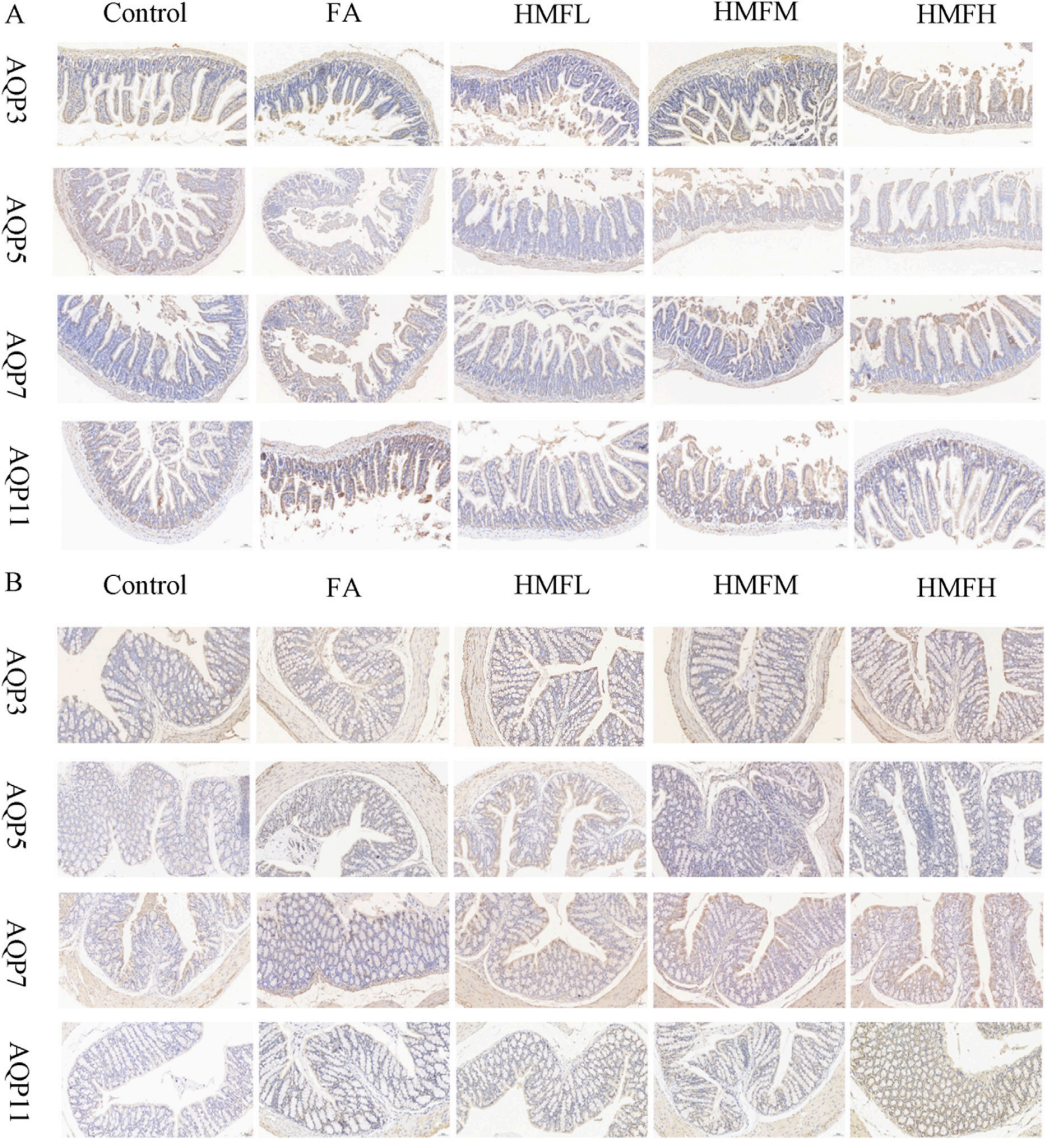
**(A)** Immunohistochemical staining results of AQP3, AQPS, AQP7 and AQP11 in small intestinal tissue; **(B)** Immunohistochemical staining results of AQP3, AQP5, AQP7 and AQP11 in colon tissue.

**FIGURE 5:**
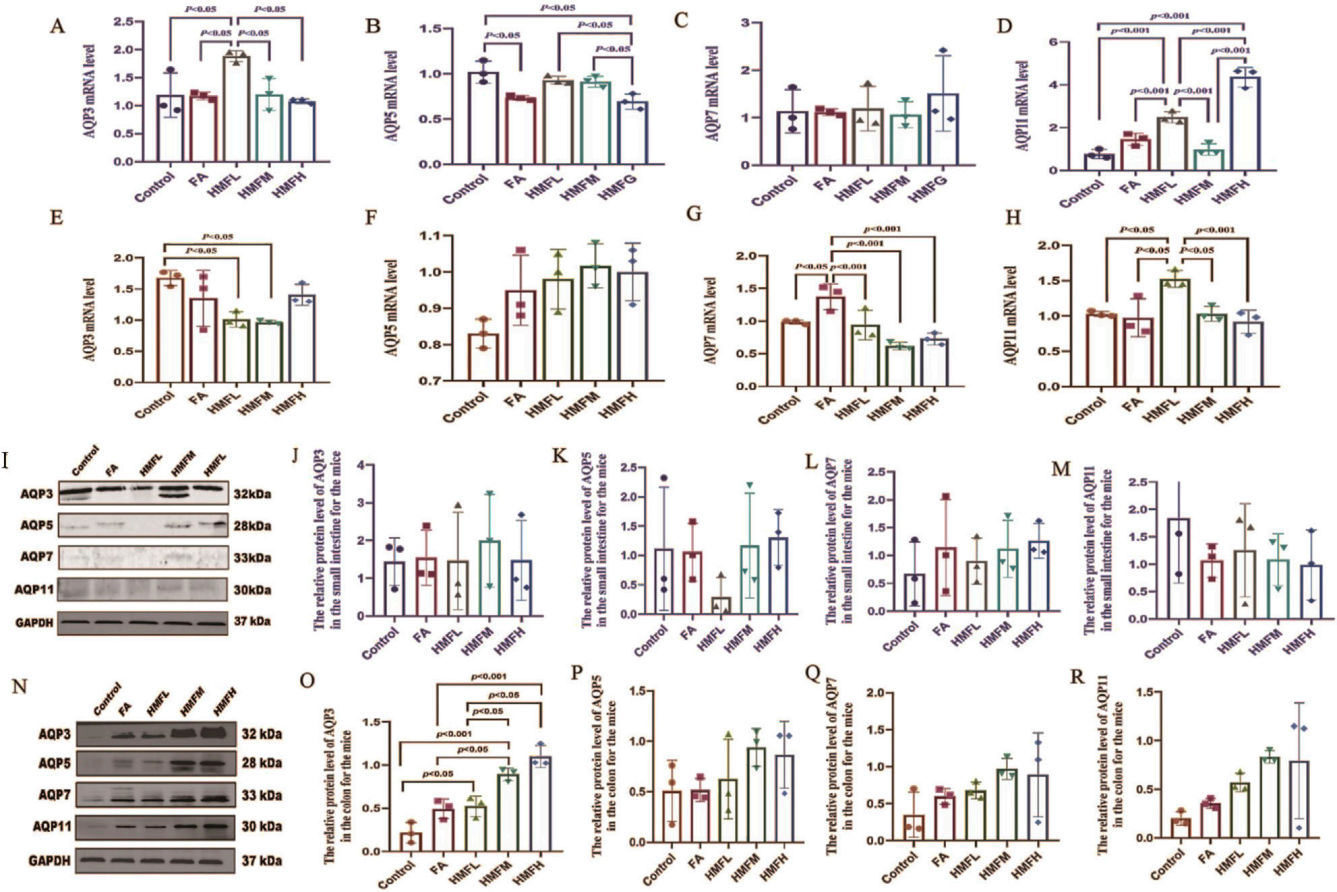
**(A–D)** mRNA levels of AQP3, AQP5, AQP7 and AQP11mRNA in the small intestine; **(E–H)** mRNA levels of AQP3, AQP5, AQP7 and AQP11mRNA in the colon; **(I–M)** protein levels in the small intestine; **(N–R)** protein levels in the colon, Note: By utilizing Image Lab to analyze the matching protein band, the re-lative expression levels of the specified proteins were qua- ntified and standardized to gapdh. It displays one of three related experiments. Data are pres- ented as the mean SEM, one-way ANO, followed the Bonferroni post hoc test, with n-3 for each group.

Immunohistochemistry analysis confirmed FA and HMF significantly elevated the expression of AQP3, AQP5, AQP7, and AQP11 in the colon and small intestine. These findings support the hypothesis that HMF can control the expression of AQPs in intestinal tissues (Figures 4A, B). Consistent with these findings, the upregulation of AQP expression triggers compensatory mechanisms in the small intestine and colon mucosa, leading to increased water absorption and subsequent dry stools. Furthermore, all dosage groups showed higher expression levels of AQP3, AQP5, AQP7, and AQP11 in both small intestine and colon tissues compared to the control group (Figures 5A–H). In conclusion, the *in vivo* animal studies demonstrate that HMF induces pathological damage to the intestinal tissues of healthy mouse. It also regulates the relative expression of AQP3, AQP5, AQP7, and AQP11 at both the mRNA and protein levels (Figures 5I–R). These alterations in AQP expression have specific effects on body fluids, including increased water intake and changes in fecal water content.

### 3.5 Effect of 3′,4′,3,5,6,7,8-heptamethoxyflavone on the expression of AQP3, AQP5, AQP7 and AQP11mRNA and proteins in CCD018Co and FHs74lnt

**FIGURE 6:**
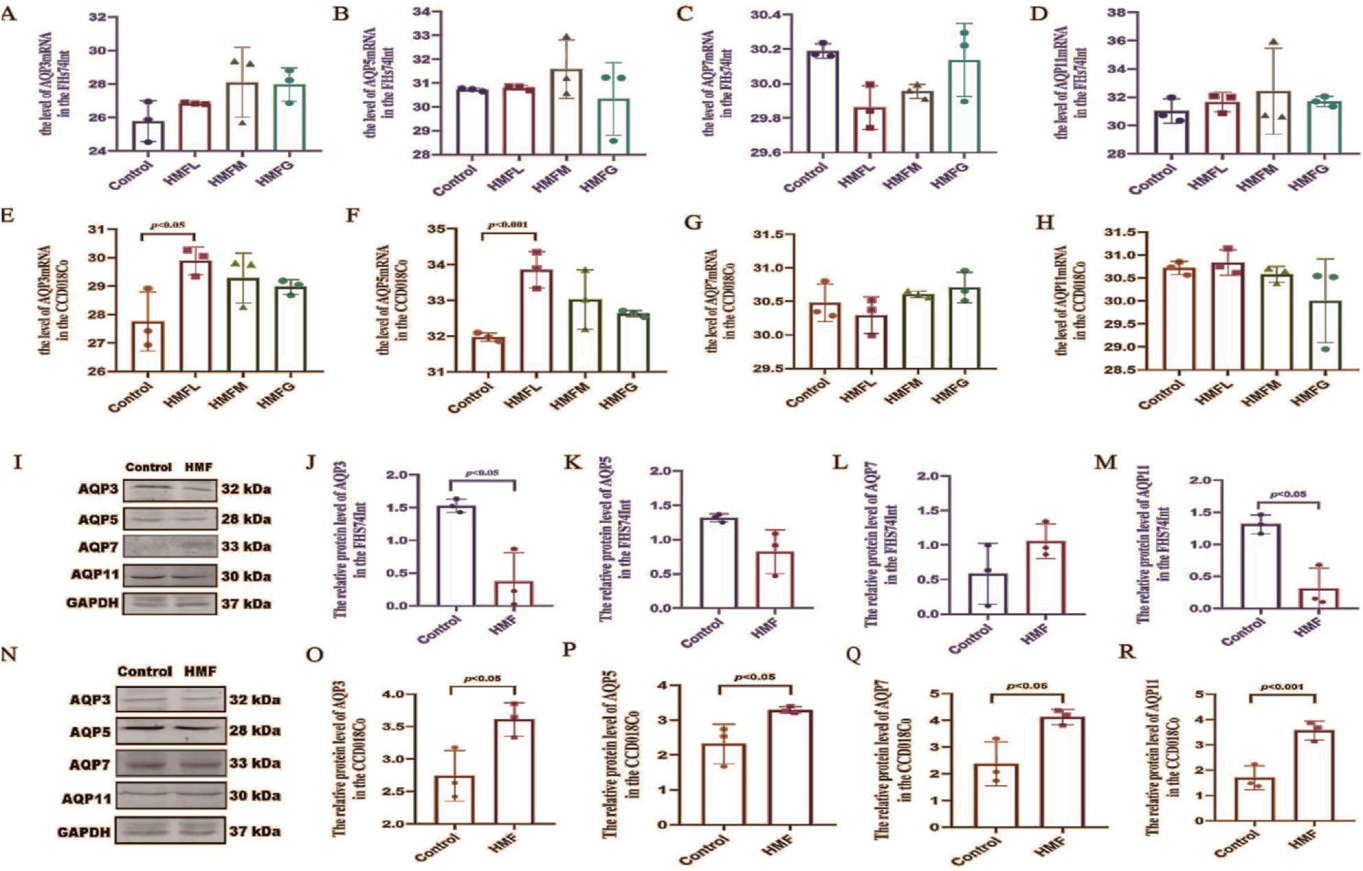
mRNA levels of AQP3, AQP5, AQP7 and AQP11 [**(A–D)** mRNA levels of the FHS74Int; **(E–H)** mRNA levels of the CCD018Co].; Proteinconcentrations as measured by western blot ana- lysis, the relative expression levels of the specified proteins were quantified and standardized to GAPDH [**(I–M)** Protein in the FHS74Int; **(N–R)** Protein in the CCD018Co].

In the *in vitro* investigations, seven different concentrations of HMF (0ug/mL, 1.25ug/mL, 2.5ug/mL, 5ug/mL, 10ug/mL, 20ug/mL, and 40ug/mL) were used. Regarding MTT assay results, HMF concentrations of 10, 20, and 40ug/mL significantly increased the survival rate of both CCD018Co and FHs74lnt cells (Supplementary Figures S1A, B). Conversely, HMF downregulated the relative expression of AQP3, AQP5, and AQP11 mRNAs in FHs74lnt cells, while upregulating the relative expression of AQP7 mRNA (Figures 6A–D). Measurements of the relative expression levels of AQPs mRNA in intestinal tissues revealed that HMF upregulated the relative expression of AQP3, AQP5, AQP7, and AQP11 mRNAs in CCD018Co cells (Figures 6E–H). Western blot assay results showed that the protein expression of AQP3, AQP5, and AQP11 in human small intestine epithelial cells was lower in the HMF group compared to the control group. However, AQP7 protein expression was higher in the HMF group (Figures 6I–M). In human colon tissue cells, the protein expression of AQP3, AQP5, AQP7, and AQP11 was higher in the HMF group compared to the control group (Figures 6N–R). The study revealed that in FHs74lnt cells, the administration of increasing concentrations of HMF led to a decrease in AQP3 content and mRNA relative expression. This decrease showed a negative correlation with the concentration of HMF. On the other hand, in CCD018Co cells, the administration of increasing concentrations of HMF resulted in a decrease in AQP3 content and mRNA relative expression, but this decrease showed a positive correlation with the concentration.

### 3.6 Effect of 3′,4′,3,5,6,7,8-heptamethoxyflavone in AQP3^−/−^ mouse

**FIGURE 7:**
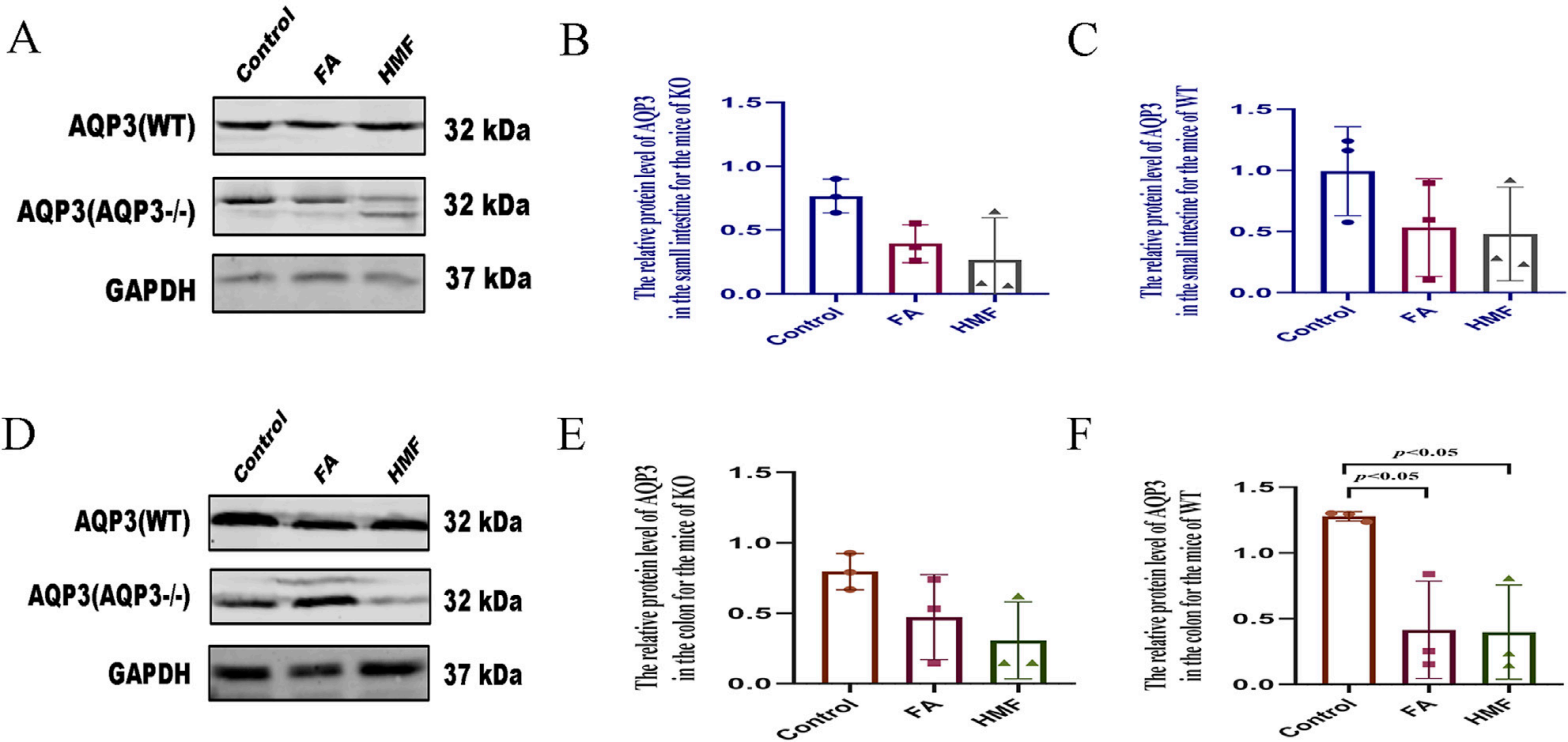
**(A–C)** The relative protein level of AQP3 in the small intestine for the mice of KO or WT; **(D–F)** The relative proteinlevel of AQP3 in the colon for the mice of KO or WT (The protein levels measured by western blot analysis. Data are expres- sed as the Mean ± SME with n = 3 every group).

This experiment was validated through Western blot and qRT-PCR analyses. In both small intestine and colon tissues, the protein expression and mRNA relative expression of APQ3 were significantly reduced after AQP3 knockdown compared to the WT group, indicating the successful construction of knockdown mouse (Figures 7A–F). Previous studies and the current analysis indicate that AQP3 protein expression plays a crucial role in regulating water distribution in normal mouse small intestine and colon tissues, particularly in water reabsorption and secretion by the intestine. The water consumption index was used as a reference to exclude individual differences. In the WT group of mouse, water consumption increased in all treatment groups compared to the control group, while in the AQP3^−/−^ group, there was no difference in water consumption after HMF administration compared to the control group (Supplementary Figures S1E, H). In the WT group, the fecal water content decreased in the FA and HMF treatment groups compared to the control group, whereas in the AQP3-KO group, there was no significant difference in fecal water content before and after treatment Supplementary Figures S1C, F). In both the WT and AQP3-KO groups, there were no significant differences in the amount of food and drink between the treatment groups and the control group, indicating that knocking out the AQP3 gene did not affect diet, appetite, feeding, or other related functions after treatment (Supplementary Figures S1D, G). Furthermore, no significant differences were observed in general indicators such as water consumption and fecal water content between the AQP3-KO group and the control group. These findings suggest that HMF, the drying metabolite of Fengyang pieces Aurantii shell, did not have a significant impact on body fluid. It is possible that the mechanism of action of HMF involves the modulation of AQP3 protein expression, which in turn affects water secretion and absorption in the body.

### 3.7 The mechanism of intestinal water channel protein regulation by 3′,4′,3,5,6,7,8- heptamethoxyflavone

**FIGURE 8:**
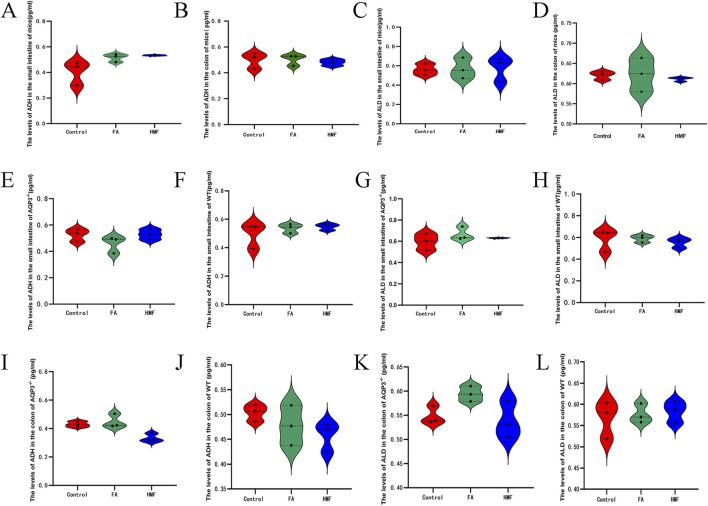
**(A, B)** The level of ADH in the the small intestine and colon for the mouse; **(C, D)** The level of ALD in the small intestine and colon for the mo- use. **(E–L)** The level of ADH and ALD in the small intestine and colon of AQP3-/- or WT.

**FIGURE 9:**
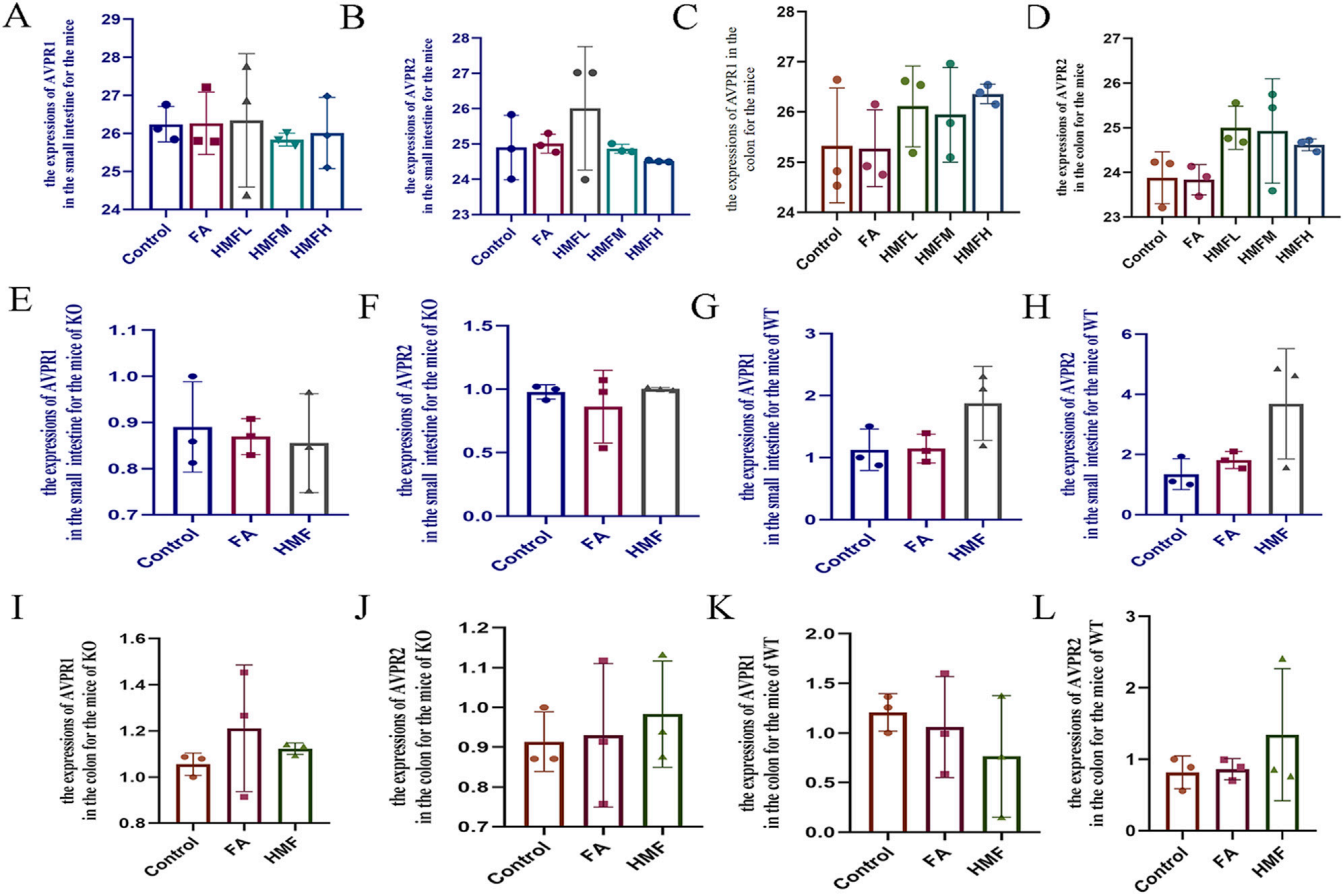
**(A–D)** The expression of AVPR1 and AVPR2 in the small intestine and colon for the mice. **(E–H)** The expression of AVPRI and AVPR2 in the small intestine for the mice of KO and WT; **(I–L)** The expression of AVPR1 and AVPR2 in the colon for the mice of KO and WT.

The levels of ALD and ADH were higher in the small intestine and colon tissues of healthy mouse in each administration group compared to the control group (Figures 8A–D). Additionally, the relative expression levels of AVPR1 and AVPR2 receptors in the small intestine and colon were higher in each administration group compared to the control group (Figures 9A–D). In AQP3 knockout mice and wild-type mice, HMF affects ADH, ALD levels and regulates AVPR1 and AVPR2 mRNA levels (Figures 8E-L; Figures 9E–L). These findings suggest that the binding of ADH and ALD to AVPR1 and AVPR2 receptors may play a role in the regulation of AQP expression. When the binding is diminished, AQP reenters the cells through endocytosis, leading to the reopening of water channels. Conversely, tighter binding of ADH and ALD to AVPR1 and AVPR2 receptors reduces the capacity to reabsorb water, resulting in increased water content in feces. Elevated levels of ALD and ADH also affect water and sodium retention in the body, thereby influencing gastrointestinal function and causing delayed digestion, stasis, bloating, and constipation.

## 4 Discussion

Among the organs involved in water-liquid metabolism, the intestinal tract plays a significant role, second only to the kidneys in absorbing and transferring water from the intake of food, thus improving nutrient supply in the body (Alessandra et al., 2016). The epithelial cells of the digestive system, including the gut, absorb water produced during the process of digestion (Zhang et al., 2007; Conner et al., 2013). A prominent water channel protein in the gut is AQP3, which is mostly found on the basolateral membrane of rat colonic epithelial cells (YanFang et al., 2014; Yupei et al., 2024; Canmin et al., 2024). When the gut is influenced by factors such as bacterial or viral infections, parasitic diseases, environmental changes, and other conditions, the expression of water channel proteins may change, leading to altered reabsorption or excessive secretion of water from the intestinal lumen (Cui et al., 2023; Ling et al., 2024). The movement of fluids across cells often involves passive diffusion and the coordinated action of AQP water channels (Huttlin et al., 2010; Kreida and Törnroth-Horsefield, 2015; Kitchen et al., 2015).

Reduced expression of AQP3 and AQP5 in the small intestine can cause intestinal inflammation, long-term mucosal damage, and an imbalance in intestinal fluid metabolism (Zhao et al., 2016; Wu et al., 2016; Meng et al., 2022). This can result in symptoms such as diarrhea and malabsorption. Disruption of the mucosal barrier function of the gut can impair the absorption and secretion processes, leading to decreased intestinal fluid levels. Polymethoxyflavones, such as HMF, have stimulating effects on the neurological and hormonal systems, increasing fluid secretion from the small intestine and promoting small intestinal fluid production. HMF induces sympathetic inhibition and vagal excitation, which further enhances the secretion of water and salts from the small intestine, as well as the production of fluids and mucus (Aleksei et al., 2022; Yu et al., 2022). These findings suggest the key role played by HMF in the regulation of intestinal fluid balance and its potential impact on gastrointestinal functions and disorders (Michael et al., 2024). The relative expression of AQP3 and its mRNA in CCD018Co cells was correlated with HMF concentration, suggesting that HMF can regulate the body’s fluid balance by influencing the role of AQP3 in intestinal water absorption and secretion. Key water channel proteins, including AQP3, AQP5, AQP7, and AQP11, are closely associated with intestinal water secretion and absorption (Li et al., 2017; Yoshimura et al., 2016). In conclusion, the relationship between HMF and APQ3 highlights the crucial role of AQP3 in regulating water absorption and secretion in intestinal tissues. These findings open up new avenues for investigating the physiological functions of AQP3 and its involvement in water homeostasis. These findings indicate that AQP3 plays a crucial role in transporting water molecules in intestinal tissues.

The regulation of intestinal water channels involves a complex interplay between neuromodulatory and hormonal factors. Various molecules, including ALD, adrenaline, and gastrin, are exchanged between intestinal mucosal epithelial cells and the body under hormonal control. These hormones play an integral role in controlling water intake and excretion by regulating ion exchange and water transport through the intestinal water channels. The kidney is considered analogous to the two bowels and is regarded as the master of water. A dysfunction in the kidney can have a significant impact on fluid transport, excretion, and the overall fluid balance within the intestines (Yu, 2015). From a contemporary medical perspective, the kidneys are vital in the body’s metabolism of water and fluids. They primarily influence the reabsorption and secretion of water and electrolytes, a process largely controlled by hormones (Muchen et al., 2022).

**FIGURE 10:**
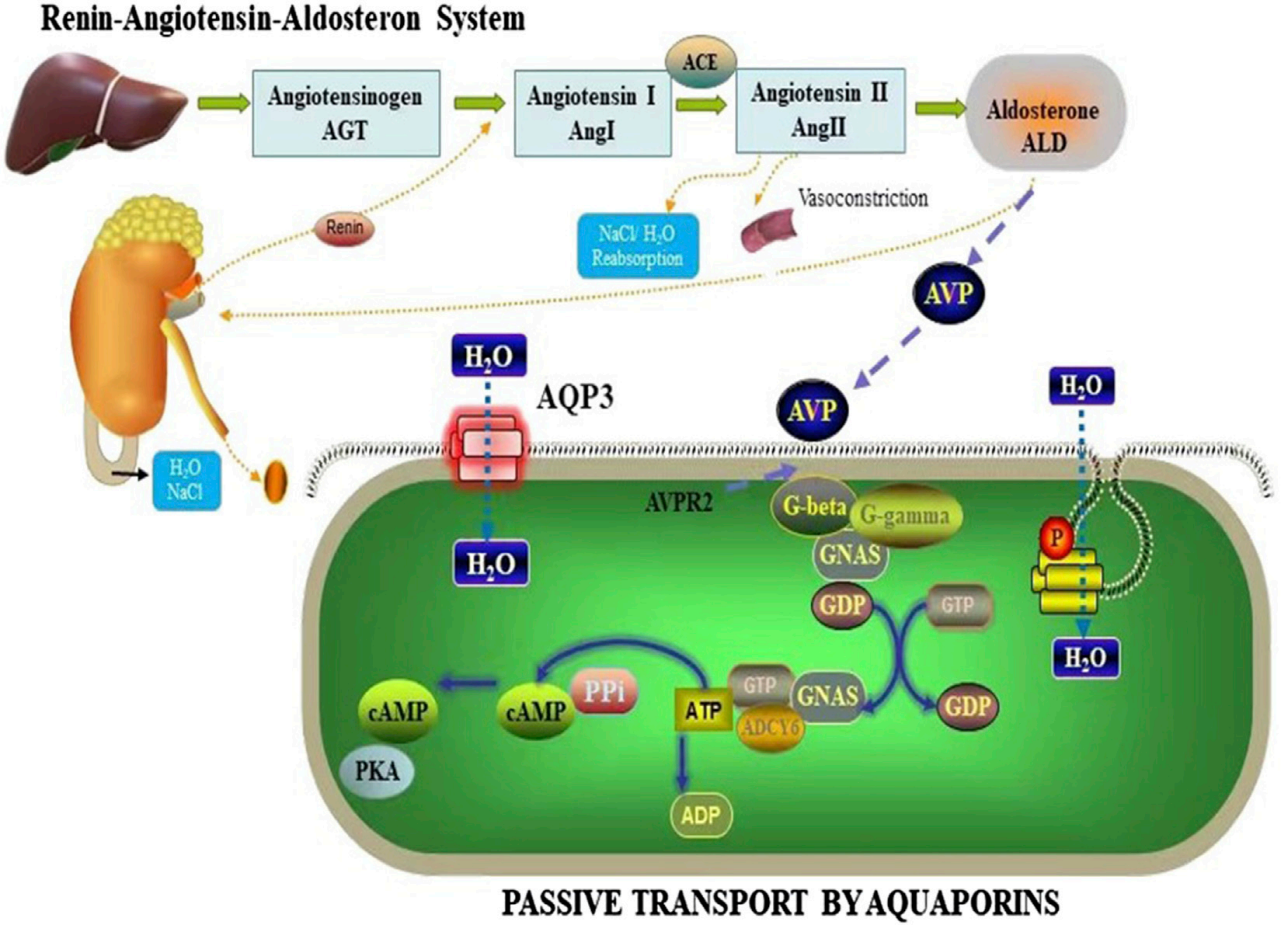
Mechanisms by which AQP3 regulates body fluids.

ADH, a key hormone secreted by the renin-angiotensin-aldosterone system (RAAS), is vital in regulating water-sodium balance and maintaining body fluid osmolality (Waanders et al., 2011; Marisa et al., 2019; Sua et al., 2021). In addition to its role in fluid balance, ADH also functions as a regulator of neurotransmitters and nerve conduction (Dingli and Hao, 2001). Studies have reported that arginine vasopressin (AVP) binds to specific receptors, including AVP1 and AVP2, which are associated with AVP-related receptor genes (Shumei et al., 2010; Linwei et al., 2023). These signaling pathways are believed to be involved in the mechanism through which AQPs regulate water secretion and uptake. The AVPR2 receptor, upon binding to ADH, activates G protein-coupled adenylate cyclase and modulates intracytoplasmic phosphatase activity (Olesen and Fenton, 2021). This signaling cascade affects cellular processes such as proliferation, differentiation, and cell death, and promotes the conversion of adenosine triphosphate (ATP) to cyclic adenosine monophosphate (cAMP), leading to the activation of protein kinases (Figure 10). Activation of this signaling cascade stimulates the production and insertion of channel proteins, including AQPs, into the apical membrane (Conner et al., 2012). Consequently, AQPs, specifically AQP3, AQP5, AQP7, and AQP11, are synthesized and regulated in the tissues of the small intestine and colon, ultimately enhancing water permeability and facilitating water transport.

The cAMP/Protein Kinase A (PKA) pathway has been implicated in the control of AQPs expression, as indicated by various studies (Meng et al., 2022). This pathway controls the production of inflammatory cytokines and activates the immune system, which is a crucial factor in irritable bowel syndrome. In constipated rats, elevated levels of cAMP and PKA were detected in the colon, leading to increased expression and activity of colonic AQPs, promoting water absorption (Chao and Zhang, 2018) and contributing to constipation. AQPs are widely expressed in the gastrointestinal tract and their expression is closely associated with the uptake and secretion of intestinal water and fluids (Liu et al., 2022; Jianghua et al., 2019; Lu et al., 2020). Wu (Dongshen et al., 2023) reported that the loss of APQ expression can significantly disrupt the metabolism of aqueous humor and affect diarrhea. Additionally, recent research has demonstrated the involvement of the cAMP/PKA pathway in the regulation of aqueous humor metabolism, with activated cAMP upregulating AQP3 expression and maintaining intestinal aqueous humor homeostasis (Chao and Zhang, 2018). Angiotensin II and ALD can activate the downstream cAMP/PKA signaling pathway through their receptors AVPR1 and AVPR2, respectively, initiating subsequent signaling pathways and regulating AQP3 expression and function (Weizhong et al., 2022). ALD can also increase water channel activity through its effects on AVP regulation. Furthermore, ALD activates the downstream phosphorylcreatine/protein kinase C (PKC) signaling pathway via its interaction with the AVPR2 receptor, thereby regulating AQP3 expression and water channel activity.

This is consistent with studies, revealing the complex mechanism of HMF in regulating water homeostasis in the body. Specifically, in mice and AQP3^−/−^, HMF not only significantly adjusted the activities of ADH and ALD, but HMF may also mediate the signaling between AVPR1 and AVPR2, which may further control the open and closed state of water channel proteins (Figure 10). When water channel proteins are in the open state, the water permeability of intestinal epithelial cells is enhanced, which promotes the rapid absorption of water and helps to maintain water homeostasis in the body under the condition of dehydration or insufficient water intake. Conversely, when water channel proteins are closed, intestinal water absorption is reduced and secretion is relatively enhanced, which helps to regulate water content in the intestine when the body is overhydrated or when excess water needs to be excreted. Thus, through this series of complex molecular mechanisms, HMF not only affects the dynamic balance of intestinal water, but may also have a profound impact on the overall water metabolism, electrolyte homeostasis, and related physiological functions of the organism. This finding provides important clues for understanding the new mechanism of water regulation in the body and potential targets for the development of novel therapeutic drugs against water metabolism-related diseases in the future.

## 5 Conclusion

Observations of water consumption and faecal water content in C57BL/6 male mice and AQP3 knockout mice revealed an imbalance of body fluid and dryness caused by HMF, which induced histopathological damage and alteration of the small intestinal and colonic tissues. Using ADH and ALD and upstream receptor genes such as AVPR1 and AVPR2 as indicators, it was clarified that HMF can affect the opening and closing of AQP3 water channels, which in turn affects water secretion and absorption.

## Data Availability

The datasets presented in this study can be found in online repositories. The names of the repository/repositories and accession number(s) can be found in the article/Supplementary Material.

## Abbreviations

ADH, Antidiuretic hormone; ALD, Aldosterone; AQP, Aquaporin; AVPR1, arginine vasopressin receptor 1; AVPR2, arginine vasopressin receptor 2; CCD018Co, Colon tissue cells; FA, FructusAurantii; FHs74lnt, Small intestinal epithelial cells; HMF, heptamethoxyflavone; qRT-PCR, Quantitative Real-time PCR.
