# Supplementary material for: Study on the effect of 3,5,6,7,8,3′,4′-heptamethoxyflavone in Fructus Aurantii by regulating intestinal aquaporin in body fluids
Source: Front Pharmacol. 2025 May 19;16:1544570. doi: 10.3389/fphar.2025.1544570 (PMC12127768; doi:10.3389/fphar.2025.1544570)
Supplement: Supplementary file 1 [file Table1.docx]

Supplementary Table 1 RT-PCR primer sequences

| Primer information | Species | Gene | Sequences |
| --- | --- | --- | --- |
| NM_008084.2 | Mouse | M-GAPDH-S  M-GAPDH-A | CCTCGTCCCGTAGACAAAATG  TGAGGTCAATGAAGGGGTCGT |
| NM_008600.5 | Mouse | AQP3-F  AQP3-R | CCTTGGCATCTTGGTGGCT  AGGAAGCACATTGCGAAGGT |
| [NM_001308641.1](https://www.ncbi.nlm.nih.gov/entrez/viewer.fcgi?db=nucleotide&id=821324786) | Mouse | AQP5-F  AQP5-R | AGAAGGAGGTGTGTTCAGTTGC  TAATGGCCGGATTGATGTGGC |
| NM_001378638.1 | Mouse | M-Aqp7(2)-S  M-Aqp7(2)-A | TGACCTGGAAGAAGTTCCCTGTA  TGCAAAGTGGTTAATGGCACC |
|  | Mouse | AQP11-F  AQP11-R | CGCCTTCGTCCTGGAGTTTC  CAACCTCACGGCACCCATT |
