# Supplementary figures and images for: Study on the effect of 3,5,6,7,8,3′,4′-heptamethoxyflavone in Fructus Aurantii by regulating intestinal aquaporin in body fluids
Source: Front Pharmacol. 2025 May 19;16:1544570. doi: 10.3389/fphar.2025.1544570 (PMC12127768; doi:10.3389/fphar.2025.1544570)

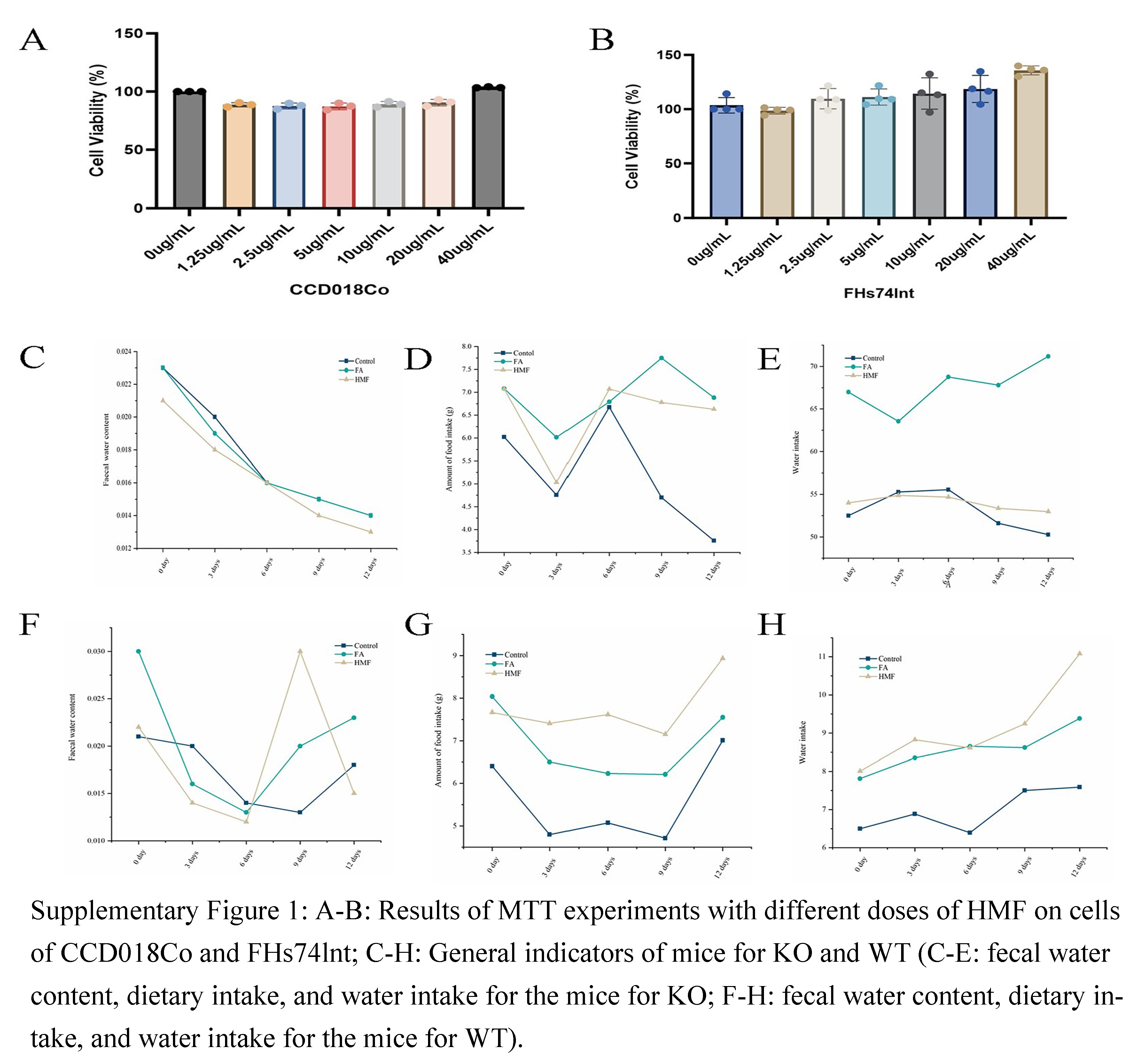

Supplement: Supplementary file 2 [file Image1.tif]
