# Supplementary material for: Study on the effect of 3,5,6,7,8,3′,4′-heptamethoxyflavone in Fructus Aurantii by regulating intestinal aquaporin in body fluids
Source: Front Pharmacol. 2025 May 19;16:1544570. doi: 10.3389/fphar.2025.1544570 (PMC12127768; doi:10.3389/fphar.2025.1544570)
Supplement: Supplementary file 3 [file Table2.docx]

Supplementary Table 2 Cell PCR Primer Information

| Name of primer | Primer sequences (5'-3') |
| --- | --- |
| Human GAPDH F | GCACCGTCAAGGCTGAGAAC |
| Human GAPDH R | TGGTGAAGACGCCAGTGGA |
| Human AQP3 F | GGGGAGATGCTCCACATCC |
| Human AQP3 R | AAAGGCCAGGTTGATGGTGAG |
| Human AQP5 F | CGGGCTTTCTTCTACGTGG |
| Human AQP5 R | GCTGGAAGGTCAGAATCAGCTC |
| Human AQP7 F | ACCCGTGGCTCCAAAATGG |
| Human AQP7 R | GGAACCAAGGCCGAATACCA |
| Human AQP11 F | TGACCCAGTATCACGTCAGC |
| Human AQP11 R | TGACCGCTTTGAGCAAGTCG |
